# Oral Manifestations of Non Vascular Ehlers‐Danlos Syndrome Cross‐Sectional Study

**DOI:** 10.1111/odi.70294

**Published:** 2026-03-16

**Authors:** Aude Grand, Benjamin P. J. Fournier, Grégoire Chevalier, Karelle Benistan, François Côme Ferré

**Affiliations:** ^1^ Health Faculty Paris Cité University Paris Île‐de‐France France; ^2^ Oral Surgery Department, APHP Charles Foix Hospital Ivry‐sur‐Seine France; ^3^ Paris Cité University and Sorbonne Paris Nord, INSERM, Santé Orale, Oral Health Montrouge France; ^4^ Reference Center of Oral and Dental Rare Diseases, Hôpital Rothschild, APHP Paris France; ^5^ Periodontal Department, APHP Charles Foix Hospital Ivry‐sur‐Seine France; ^6^ AP‐HP, GHU Paris Saclay CHU Raymond Poincaré, Reference Center for Non Vascular Ehlers‐Danlos Syndromes Garches France; ^7^ INSERM, UMR1179, University of Versailles Saint Quentin en Yvelines, UFR Simone Veil‐Santé Montigny‐le‐Bretonneux France

**Keywords:** frenulum, molecular diagnostic, non vascular Ehlers Danlos syndrome, oral diseases, temporomandibular disorders

## Abstract

**Background:**

Ehlers–Danlos syndromes are rare hereditary connective tissue disorders; however, their oral manifestations remain poorly characterized in molecularly confirmed individuals. The aim of this study was to describe the oral phenotype of patients with non‐vascular Ehlers‐Danlos syndromes compared to healthy controls.

**Methods:**

In this single‐center, cross‐sectional study, 14 patients molecularly diagnosed with non‐vascular Ehlers‐Danlos syndromes and 30 matched healthy controls underwent a complete clinical and radiological oral examination. Data were compared using chi‐squared, Fisher's exact, Student's *t*‐tests, or Wilcoxon tests as appropriate.

**Results:**

Compared to controls, patients with non‐vascular Ehlers‐Danlos syndromes had a significantly higher prevalence of temporomandibular disorders (64.3% vs. 16.7%; *p* = 0.004), gingivitis (85.7% vs. 56.7%; p = 0.017), and lingual parafunction (57.1% vs. 16.7%; *p* = 0.012). Anatomical variations were also more frequent, including atrophy of the lingual (85.7% vs. 10.0%; *p* < 0.001) and inferior labial frenula (78.6% vs. 20.0%; *p* < 0.001), pulpal retractions (61.5% vs. 27.6%; *p* = 0.047), and dental root hypoplasia (57.1% vs. 20.0%; *p* = 0.034).

**Conclusion:**

Our findings suggest that patients with non‐vascular Ehlers‐Danlos syndromes present a distinct pattern with oral manifestations. These findings, including notably frenula atrophy and specific dental anomalies. These oral findings commonly observed in non‐vascular Ehlers‐Danlos syndromes patients can aid in diagnosis and underscore the need for specialized dental care.

## 1 Introduction

Ehlers‐Danlos syndromes (EDS) are a group of rare hereditary disorders whose overall prevalence is estimated at 1/5000 for all types combined (Kapferer‐Seebacher et al. 2017). Their transmission is usually autosomal dominant or autosomal recessive. According to the 2017 international New York classification, 13 EDS subtypes are currently recognized, 12 of which have an identified genetic origin. They are caused by variants in genes encoding connective‐tissue proteins, leading to defects in the synthesis, assembly, or stability of collagens and, consequently, to connective‐tissue abnormalities. The clinical spectrum is broad, ranging from mild joint hypermobility to life‐threatening complications depending on the subtype (Malfait et al. 2017).

Generally, the different subtypes present with overlapping clinical manifestations. Prognosis and mortality vary significantly across the different forms. For example, the vascular type (vEDS), which is not the subject of this study, is associated with a high risk of mortality due to arterial or organ rupture (Malfait et al. 2017). The non‐vascular forms present a different prognosis but still cause significant morbidity, with common systemic manifestations including skin hyperextensibility, joint hypermobility, abnormal scarring, and chronic musculoskeletal pain. However, life‐threatening complications are not entirely absent from this group; spontaneous arterial ruptures have been reported in other forms such as kyphoscoliotic EDS (kEDS) and classic EDS (cEDS), which can alter the prognosis (Malfait et al. 1993).

As with all rare diseases, patient care in France is organized through labeled reference centers. Patients with the vascular EDS variant are followed up at dedicated vascular‐rare‐disease reference centers, while all other forms are managed by centers dedicated to non‐vascular EDS.

Among the non‐vascular forms, the classical type (cEDS) is one of the most common, with an estimated prevalence of 1/30,000 (Centre de référence MOC/Filière OSCAR 2020). In most cases, the causal mutation involves the type V collagen genes *COL5A1* or *COL5A2*, altering collagen structure, stability, and assembly, and resulting in skin hyperextensibility, skin fragility with atrophic scars, and joint hypermobility.

Two other subtypes, which are much less common, are the classical‐like form (clEDS) and the periodontal form (pEDS); their prevalence is still unknown (Malfait et al. 2017). clEDS results from pathogenic variants in *TNXB*, which encodes tenascin‐X, an extracellular matrix glycoprotein interacting with fibrillar collagens (notably type I) and regulating the distance between collagen fibrils. Mutations in the *TNXB* gene lead to a phenotype similar to cEDS but without atrophic scarring (Malfait et al. 2017). pEDS is caused by mutations in *C1R* or *C1S*, encoding the C1 complement subunits; it is characterized by early‐onset severe periodontitis, lack of attached gingiva, and pretibial brown plaques corresponding to hemosiderin deposits (Kapferer‐Seebacher et al. 2021). Another subtype included in this group, kEDS, is primarily characterized by congenital muscle hypotonia, early‐onset kyphoscoliosis, and marked joint laxity (Malfait et al. 2017).

Patients affected with EDS report an impaired oral‐health‐related quality of life (Hanisch et al. 2020; Balke et al. 2023). Several studies have described oral manifestations across all EDS subtypes (Kapferer‐Seebacher et al. 2017; Lepperdinger et al. 2021; Mitakides and Tinkle 2017) which may play a potential role in raising clinical suspicion. However, the lack of molecular diagnosis in most of these studies markedly limits the validity of their conclusions. Other conditions, such as the hypermobility spectrum disorders, show overlapping clinical features and can be difficult to distinguish from the hypermobile EDS subtype, for which no genetic cause has yet been identified (Yew et al. 2021).

As EDS is a rare condition with which dental practitioners are often unfamiliar, recognizing oral findings could help to establish clinical suspicion and facilitate an earlier diagnosis, particularly during routine dental care appointments. Therefore, it is essential to systematically document oral manifestations in genetically confirmed patients to improve diagnostic accuracy and patient management. The aim of this study was to provide a comprehensive oral description of a group of molecularly confirmed non‐vascular EDS patients and to compare these findings with those of a matched control group. We hypothesized that a systematic assessment of dental, gingival, and osteo‐articular characteristics would reveal EDS‐specific features.

## 2 Materials and Methods

### 2.1 Study Design

This cross‐sectional study was designed according to guidelines of the STROBE statement. Molecularly diagnosed patients with non‐vascular Ehlers‐Danlos syndromes (nvEDS) and healthy patients were included retrospectively from January 2021 to March 2023 and prospectively from April to September 2023 in a single center (Figure 1). All participants underwent a full oral examination. Physicians were aware of participants' status at inclusion.

The inclusion criteria were EDS patients with a molecular diagnosis established at the nvEDS reference center and who had agreed to an oral examination at Charles‐Foix Hospital. The exclusion criteria were patients who refused to participate in the study, patients who did not have a panoramic X‐ray, and patients who had a history of frenectomy.

Standardized clinical observations of the teeth and surrounding tissues were carried out by physicians experienced in EDS. A standard orthopantomogram was obtained from each participant to evaluate dental disease, bone structures, tooth position and shape, and the temporomandibular joint (TMJ).

The study was approved by the Sorbonne University research ethics committee (CER‐2023‐GRAND‐ManOraSED) and all patients gave written informed consent.

### 2.2 Study Population

Genetically diagnosed nvEDS patients followed at the French Reference Center for Non‐Vascular EDS (Raymond‐Poincaré Hospital, AP‐HP, Garches) were referred to the dental department of Charles‐Foix Hospital for a systematic oral check‐up. Controls were recruited among consecutive healthy subjects attending the same department for routine care. None had suspected connective‐tissue disorder, and their medical history was minimal. Control subjects were age‐ and sex‐matched to nvEDS patients at an approximate 2:1 ratio.

### 2.3 Medical and Genetic Data

All nvEDS patients were clinically assessed by senior physicians of the Reference Centre for Non‐Vascular EDS. Personal history and clinical features were documented on a standardized form; major and minor diagnostic criteria were recorded according to the 2017 New York classification. Only patients with genetic confirmation were included (Table 1).

**TABLE 1:** Genetic characteristics of the 14 non‐vascular Ehlers‐Danlos syndrome patients.

Patient	Age (years)	Sex (M/F)	Gene	DNA	Protein
Classical EDS	64	F	COL1A1	Exon 14 c.934 C>T	p.Arg312Cys
Classical EDS	37	F	COL5A1	Exon 27 c.2374 C>T	p.Arg792*
Classical EDS	63	M	COL5A1	Exon 27 c.2374 C>T	p.Arg792*
Classical EDS	43	F	COL5A1	Exon 27 c.2374C>T	p.Arg792*
Classical EDS	55	F	COL5A1	Exon 63 c.5013 C>A	p.Cys1671*
Classical EDS	33	M	COL5A1	Exon 51 c.4050dup	p.Gly1351Argfs*14
Classical EDS	27	M	COL5A1	Exon 39 c.3014_3030del	p.Thr1005Asnfs*3
Classical EDS	68	F	COL5A1	Exon 15 c.1727dup	p.Gly577Trpfs*39
Classical EDS	60	M	COL5A2	Exon 8 c.3445 G>T	p.Gly1149Cys
Classical‐like EDS	26	F	TNXB	Exon 40 c.12186_12187del c.10922‐?_c.12729 +?del (deletion exons 33_44)	p.Arg4063Trpfs*40 Unknown
Classical‐like EDS	30	M	TNXB	Exon 35 c.(11,380 + 1_11381–1)_(11,524 + 1_11525–1)del	Unknown
Classical‐like EDS	28	M	TNXB	Exon 35 c.(11,380 + 1_113811)_(11,524 + 1_11525–1)del	Unknown
Kyphoscoliotic EDS	17	M	FKBP14	c.362dupC	p.Glu122Argfs*7
Periodontal EDS	25	M	C1S	Exon 8 c.961 T>G	p.Cys321Gly

*Note:* Classical EDS and Periodontal EDS are autosomal dominant diseases. Classic‐like EDS and kyphoscoliotic EDS are autosomal recessive diseases.

### 2.4 Oral Examination

The oral examination included both clinical and radiological examinations. Due to technical limitations, the clinical examination was performed once per participant by two experienced practitioners working in tandem, with systematic photographic documentation. Before the study began, the two examiners trained together on the radiological criteria described below by calibrating their assessments on a set of practice radiographs to ensure consistent interpretation. Inter‐examiner calibration was conducted using 15 control patients for the following variables: pulp retraction, dental root fusion, exceeding root length, root hypoplasia, pulp stones, and TMJ remodeling. For all variables, kappa values ranged from 0.96 to 1.00. Radiographic analysis was performed independently by the two trained and experienced examiners. Any disagreement was resolved through joint review.

#### 2.4.1 Periodontal and Mucosal Assessment

The periodontal data consisted of standardized measurement of gingival fragility, thickness, inflammation, and labial/lingual frenulum size. Bleeding on probing (BOP) was assessed with a periodontal probe and graded according to Löe & Silness (Loe and Silness 1963). Gingival thickness was measured by the ability to see the periodontal probe through gingival tissue (Järveläinen et al. 2009). Gingival inflammation was classified with the 2018 consensus thresholds: BOP < 10% = healthy; 10%–30% = localized gingivitis; > 30% = generalized gingivitis (Trombelli et al. 2018). Periodontal disease was diagnosed clinically and radiographically using the 2017 Chicago Classification (Caton et al. 2018). The threshold for the lack of attached gingiva was set at 2 mm (Scheyer et al. 2015). Lingual, superior, and inferior labial frenula were examined by an experienced periodontist and classified as restrictive, physiological, atrophic or absent.

#### 2.4.2 Occlusal‐Functional Assessment

History of orofacial pain originating either from the masticatory muscles or the joint capsule was recorded, including reports of pain in the jaw, temples, face, pre‐auricular area or the TMJ both at rest and in function. Physical examination included the observation and measurements of mandibular motion (maximal interincisal opening, lateral movements and protrusion), palpation of the masticatory muscles (masseter, temporalis, medial and lateral pterygoid muscles) and static and dynamic TMJ palpation. Panoramic radiographs were examined for marked flattening or sclerosis of articular surfaces. Joint hypermobility was defined as mouth opening ≥ 50 mm according to previous studies (De Coster et al. 2005; Zawawi et al. 2003). Temporomandibular disorders (TMD) were classified according to the modified Diagnostic Criteria for Temporomandibular Disorders (DC/TMD): group I (muscular), II (disc displacement), III (arthralgia/arthritis/arthrosis), IV (TMJ hypermobility), V (tension‐type headache) (Schiffman et al. 2014). These clinical indicators make it possible to characterize each patient's TMD precisely, and patients may belong to one or more groups in this classification.

Separately, headaches were diagnosed according to the International Classification of Headache Disorders, 3rd edition (ICHD‐3) (*Cephalalgia: An International Journal of Headache* 2018).

Lingual parafunctions (low resting tongue position, tongue interposition, or atypical swallowing with tongue thrust) were also recorded (Kamdar and Al‐Shahrani 2015).

#### 2.4.3 Dental Assessment

Teeth were assessed clinically and radiologically to detect structural anomalies and dental lesions (caries, traumatic lesions). Root fusion and pulp‐chamber size were assessed on orthopantomograms. Root length was judged normal when longer than the crown but less than twice its length; excessively long roots (radiculomegaly) had a crown/root ratio > 2 (Hölttä et al. 2004), short roots (hypoplasia) ≤ 1 (Luder 2015). Pulpal retraction was recorded when at least one pulp horn was receded (teeth with restorations and pulpal retraction were excluded from this analysis) (Smith et al. 2001). The decayed–missing–filled teeth (DMFT) index was calculated on 28 teeth (third molars excluded) (Klein et al. 1938). Missing teeth due to agenesis were counted as missing.

For root fusion analysis, only maxillary first molars (De Coster et al. 2005; Royer and Han 2022) and mandibular first and second molars (Jannati et al. 2019; Jones 1984; Lepperdinger et al. 2022; Jensen and Storhaug 2011) were considered; fusion of third molars and maxillary second molars was disregarded because of its frequency in the general population (Kim et al. 2012).

#### 2.4.4 Statistical Analysis

Qualitative variables were described as number (percentage); quantitative variables as median (inter‐quartile range). Pearson's *χ* ^2^ or Fisher's exact test compared categorical data; Student's *t*‐test or Wilcoxon's test compared continuous data. *p*‐values were two‐sided; significance was set at 0.05. 95% confidence intervals (CI) are provided. For binary comparisons, odds ratios.

(OR) and 95% confidence intervals were computed from 2 × 2 contingency tables. Fisher's exact test (or chi‐square test) was used to assess statistical significance. No multivariable regression was performed. Because of the exploratory nature of this study and the rarity of the condition, no a priori sample size calculation was performed. The analyses were based on all eligible patients. All the computations were performed with R++ and Graphpad Prism software.

Some data were missing for TMJ remodeling (2 EDS and 2 controls) because not all of the TMJ was visible on the orthopantomogram. Data were also missing for pulpal retractions (1 EDS and 1 control), root fusion (1 control), and pulp stones (1 EDS and 3 controls); the analysis was uninterpretable due to pulpal restorations or missing teeth. In all cases, the statistical analyses were performed on the total number of patients minus those with missing data. For these 4 categories, the number of patients analyzed is indicated by the “*n*” in the corresponding rows (Tables 2 and 3).

**TABLE 2:** Temporomandibular joint characteristics of patients with nvEDS and healthy controls.

Variable	nvEDS patients *n* (%) or mean (SD)	Controls *n* (%) or mean (SD)	*p*	OR
TMD group I: muscle disorders				
Myofascial pain (MP)	7 (50.00%)	1 (3.33%)	0.000597875	29 [3.05; 275.69]
MP with limitation	1 (7.14%)	0 (0.00%)	NS	
MP with referal	2 (14.29%)	0 (0.00%)	NS	
Temporal tendonitis	0 (0.00%)	0 (0.00%)	NS	
TMD group II: disc displacements				
Disc displacement with reduction	2 (14.29%)	4 (13.33%)	NS	
Disc displacement with reduction with intermittent locking	1 (7.14%)	0 (0.00%)	NS	
Disc displacement without reduction	0 (0.00%)	0 (0.00%)	NS	
TMD group III: Arthralgia, Osteoarthritis, Osteoarthrosis				
Arthralgia	1 (7.14%)	1 (3.33%)		
TMJ osteoarthritis	0 (0.00%)	0 (0.00%)		
TMJ osteoarthrosis	0 (0.00%)	0 (0.00%)		
TMD group IV: TMJ hypermobility				
Luxation/subluxation	3 (21.43%)	0 (0.00%)	0.0274841	
TMD group V: Tension‐type headache with temporal muscle sensitivity				
Few episodes	2 (14.29%)	1 (3.33%)	NS	
Frequent episodes	0 (0.00%)	0 (0.00%)		
Chronic headaches	0 (0.00%)	0 (0.00%)		
Total TMD	9 (64.29%)	5 (16.67%)	0.00399867	9 [2.10; 38.56]
Hypermobility	5 (35.71%)	3 (10.00%)	NS	5 [0.99; 25.21]
Bruxism	1 (7.14%)	5 (16.67%)	NS	0.38 [0.04; 3.65]
Cracking	6 (42.86%)	4 (13.33%)	0.041	4.88 [1.10; 21.69]
Dislocation episode	3 (21.43%)	0 (0.00%)	0.0274841	
Headaches	6 (42.86%)	1 (3.33%)	0.00244052	21.75 [2.28; 207.82]
Lingual parafunction	8 (57.14%)	5 (16.67%)	0.0116881	6.67 [1.60; 27.83]
TMJ remodeling	5 (41.67%) (*n* = 12)	6 (21.43%) (*n* = 28)	NS	

**TABLE 3:** Dental characteristics of patients with nvEDS and healthy controls.

Variable	nvEDS patients *n* (%) or mean (SD)	Controls *n* (%) or mean (SD)	*p*	OR
Pulp retraction				
Total pulp retraction	8 (61.54%) (*n* = 13)	8 (27.59%) (*n* = 29)	0.0472135	4.20 [1.05; 16.73]
Pulp retraction on at least 7 teeth	6 (46.15%) (*n* = 13)	1 (3.45%) (*n* = 29)	0.0019082	24 [2.47; 233.06]
Root fusion	5 (35.71%)	8 (27.59%) (*n* = 29)	NS	1.46 [0.37; 5.70]
Exceeding root length	2 (14.29%)	4 (13.33%)	NS	1.08 [0.17; 6.75]
Root hypoplasia				
Total root hypoplasia	8 (57.14%)	6 (20.00%)	0.034095	5.33 [1.33; 21.33]
Root hypoplasia on at least 7 teeth	4 (28.57%)	1 (3.33%)	0.0294952	11.6 [1.16; 116.42]
Pulp stones	9 (69.23%) (*n* = 13)	13 (48.15%) (*n* = 27)	NS	2.42 [0.60; 9.82]
Dental agenesis	1 (7.14%)	0 (0.00%)	NS	

## 3 Results

### 3.1 Participants

From January to September 2023, 18 molecularly confirmed nvEDS patients followed at the Reference Center were approached; 4 declined. Ten nvEDS patients were therefore included: 9 were affected with classical EDS, 3 with classical‐like EDS, 1 with kyphoscoliotic EDS, and 1 with periodontal EDS. Forty‐three control subjects were screened for inclusion at a ratio of 2:1. General characteristics are shown in Table 4.

**TABLE 4:** Baseline characteristics of patients with nvEDS and healthy controls.

Variable	nvEDS patients *n* (%) or mean (SD)	Controls *n* (%) or mean (SD)	*p*	OR
*N*	14 (100%)	30 (100%)	—	—
Age	41,14 (17.38)	40,1 (18.94)	NS	—
Sex				
Male	8 (57.14%)	14 (46.67%)	NS	—
Female	6 (42.86%)	16 (53.33%)	NS	—
EDS type				
Controls	0 (0%)	30 (100%)	0.00049975	—
Classical	9 (64.29%)	0 (0%)		—
Classical‐like	3 (21.43%)	0 (0%)		—
Kyphoscoliotic	1 (7.14%)	0 (0%)		—
Periodontal	1 (7.14%)	0 (0%)		—
Smoking			NS	—
No	12 (85.71%)	25 (83.33%)	NS	—
Active smoking	2 (14.29%)	2 (6.67%)	NS	—
Smoking cessation	0 (0%)	3 (10.00%)	NS	—
Previous dentofacial orthodontic treatment	6 (42.86%)	7 (23.33%)	NS	2.46 [0.64; 9.55]
DMFT index	5.29 (6.39)	8.17 (6.77)	NS	
Skin and joint characteristics				
Joint hypermobility	13 (92.86%)	0 (0%)	2.96889 × 10^−10^	
Skin hyperextensibility	13 (92.86%)	0 (0%)	2.96889 × 10^−10^	
Facial ecchymoses	1 (7.14%)	0 (0%)	NS	
Pretibiales plaques	1 (7.14%)	0 (0%)	NS	

### 3.2 Periodontal Status

EDS patients were significantly more likely to have gingivitis than the control group (85.71% vs. 56.67%). No significant difference was observed between groups for gingival index, periodontitis prevalence, gingival thinness or lack of attached gingiva (Table 5). 100% of the nvEDS patients with thick gingiva were of the classical subtype.

**TABLE 5:** Periodontal characteristics of patients with nvEDS and healthy controls.

Variable	nvEDS patients *n* (%) or mean (SD)	Controls *n* (%) or mean (SD)	*p*	OR
Gingival index	0.99 (0.66)	0.97 (0.85)	NS	
Bleeding on Probing (BOP)	0.39 (0.23)	0.54 (1.04)	NS	
Thin gingiva	7 (50.00%)	16 (53.33%)	NS	
Lack of attached gingiva	4 (28.57%)	10 (33.33%)	NS	
Gingival inflammation	12 (85.71%)	17 (56.67%)	NS	4.59 [0.87; 24.18]
Periodontitis stage				
Healthy periodontium	7 (50.00%)	12 (40.00%)	NS	
Periodontitis stage 1	3 (21.43%)	5 (16.67%)		
Periodontitis stage 2	3 (21.43%)	5 (16.67%)		
Periodontitis stage 3	1 (7.14%)	8 (26.67%)		
Periodontitis grade				
Periodontitis grade A	3 (42.86%)	12 (66.67%)	NS	
Periodontitis grade B	3 (42.86%)	4 (22.22%)		
Periodontitis grade C	1 (14.29%)	2 (11.11%)		
Gingival diagnosis				
Gingival health	2 (14.29%)	13 (43.33%)	0.0174913	
Localized gingivitis	2 (14.29%)	10 (33.33%)		
Generalized gingivitis	10 (71.43%)	7 (23.33%)		
Lingual frenulum atrophied or absent	12 (85.71%)	3 (10.00%)	0.00049975	
Superior labial frenulum atrophied	7 (50.00%)	7 (23.33%)	NS	0.30 [0.08; 1.170]
Inferior labial frenulum atrophied	11 (78.57%)	6 (20.00%)	0.000508848	0.67 [0.12; 0.324]

**FIGURE 1:**
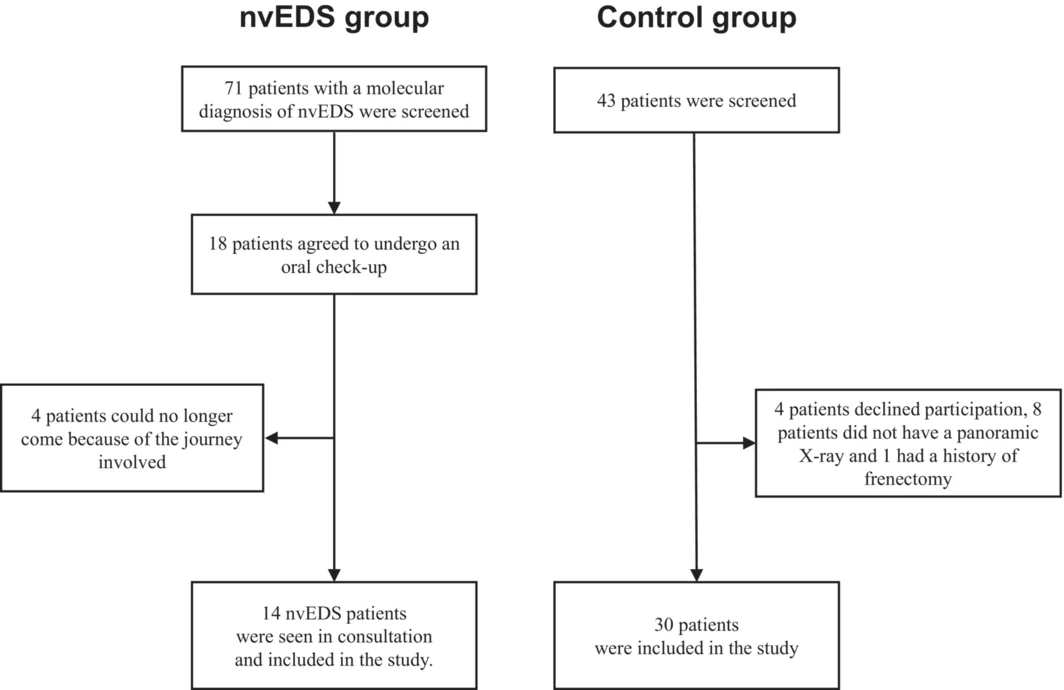
Flow‐chart.

Atrophic lingual and inferior labial frenula were significantly more frequent in nvEDS patients. An atrophic or absent lingual frenulum was found in 85.7% of nvEDS patients (*n* = 12) versus 10% of controls (*n* = 3) (Figure 2A,B). Inferior labial frenulum atrophy occurred in 78.6% of nvEDS patients versus 20% of controls (*n* = 6) (Figure 2C). No significant difference was found for the superior labial frenulum (Table 5).

**FIGURE 2:**
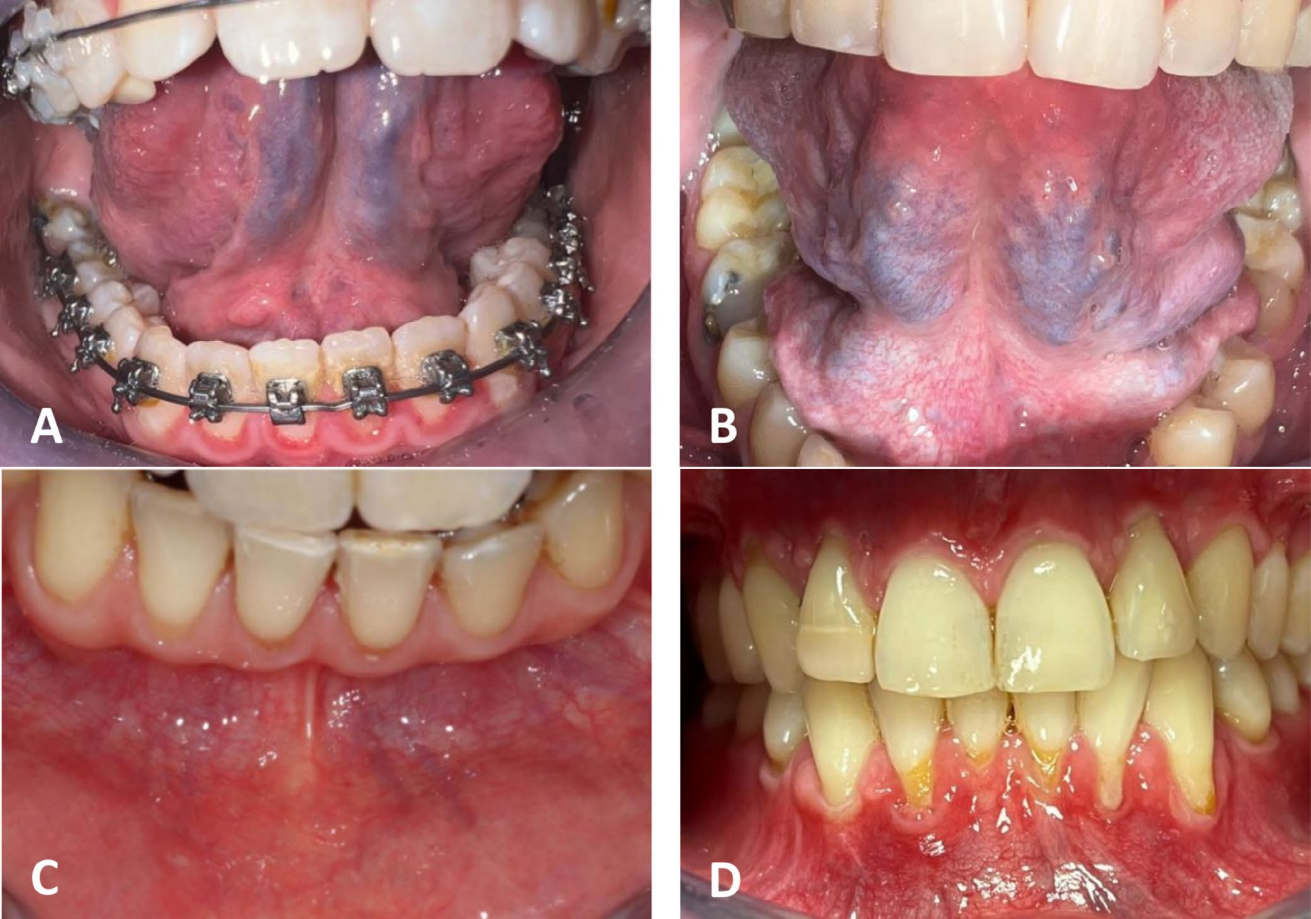
Periodontal characteristics of patients with nvEDS. (A) Absence of a lingual frenulum in a kyphoscoliotic EDS patient. (B) Absence of a lingual frenulum in a classic EDS patient. (C) Atrophy of the inferior labial frenulum in a classic‐like EDS patient. (D) Lack of attached gingiva in a periodontal EDS patient.

### 3.3 Temporomandibular‐Joint Status

We found a significantly higher proportion of TMDs in nvEDS patients (64.3%, *n* = 9) than controls (16.7%, *n* = 5). Group I (myofascial pain) disorders were significantly more common in nvEDS (50.0% (*n* = 7) vs. 3.3% (*n* = 1)), as were group IV disorders (TMJ hypermobility: 21.4% vs. 0%). A past history of true TMJ dislocation was also more frequent (21.4% (*n* = 3) vs. 0% (*n* = 0)). Headaches were significantly more common in nvEDS (42.9% (*n* = 6) vs. 3.3% (*n* = 1)). Specifically, the nvEDS group included 4 cases of migraine and 2 cases of tension‐type headache, the control group included 1 case of tension‐type headache. Lingual parafunctions (57.1% (*n* = 8) vs. 16.7% (*n* = 5)) were also significantly more common in the nvEDS group. No radiographic difference in TMJ remodeling was detected (Table 2). There was no difference between groups in the prevalence of a high‐arched palate.

### 3.4 Dental Status

Short roots were significantly more frequent in nvEDS patients (57.1%, *n* = 8) than in controls (20.0%, *n* = 6) (Figure 3A). Pulpal retraction was also more common (61.5%, *n* = 8 vs. 27.6%, *n* = 8) (Figure 3B). No significant differences were seen for pulp stones, radiculomegaly, or tooth agenesis (Table 3).

**FIGURE 3:**
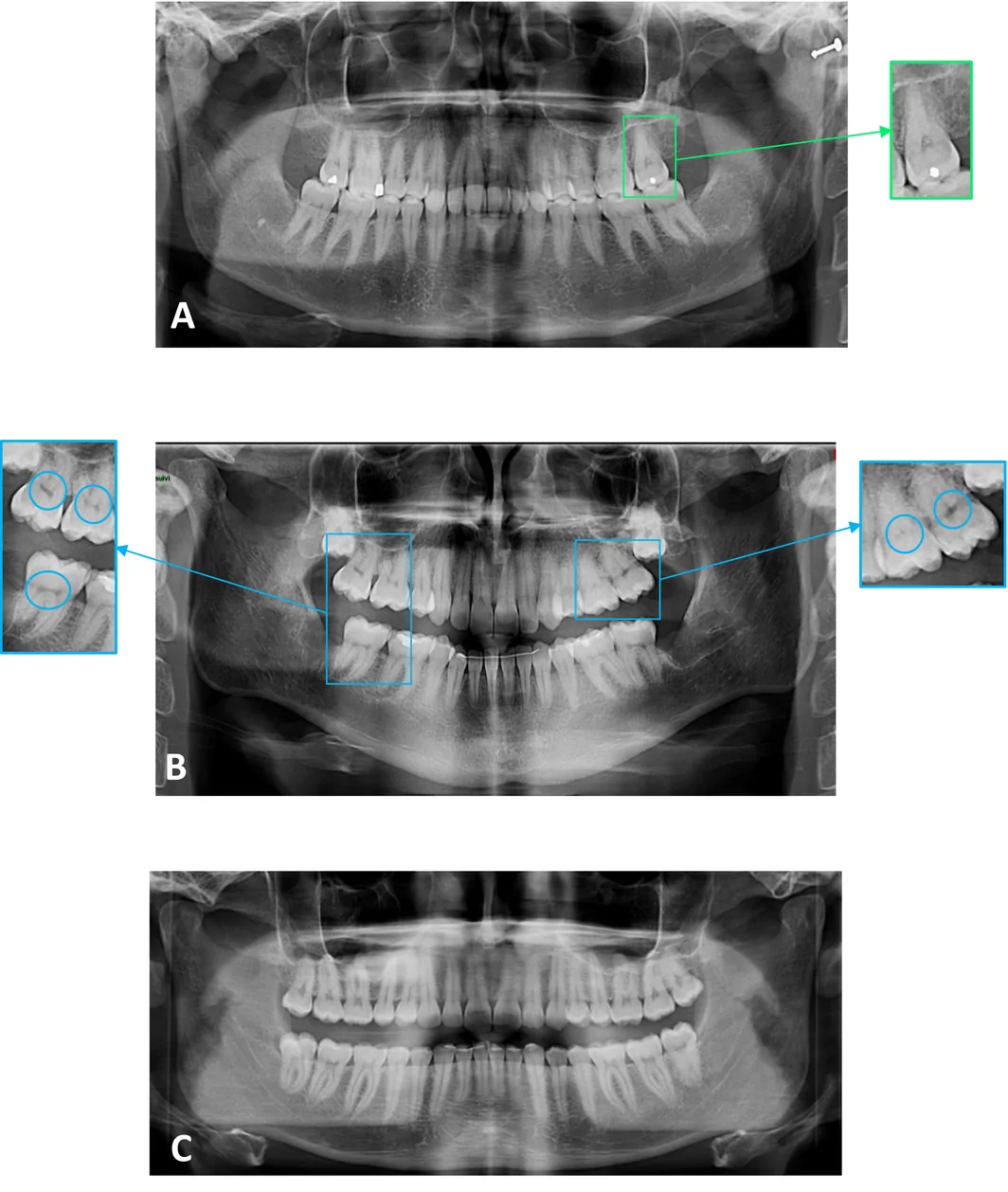
Dental characteristics of patients with nvEDS. (A) Orthopantomogram of a 37‐year‐old female patient with classic EDS showing hypoplasia of the roots of the molars associated with the presence of pulpoliths. (B) Orthopantomogram of a 29‐year‐old classical‐like EDS patient showing pulpal retractions on almost all the teeth. (C) Orthopantomogram of a patient in the control group.

## 4 Discussion

This cross‐sectional study is one of the largest oral studies in molecularly confirmed nvEDS to date.

The oral features seen in these nvEDS patients seem to reflect an overall fragility of the extracellular matrix (ECM) rather than separate or unrelated dental problems. This is biologically plausible, as fibrillar collagens (types I, III, and V) and ECM regulators like tenascin‐X work together to maintain the strength and flexibility of mucosa, ligaments, and dentin throughout growth and repair (Malfait et al. 2017). When these proteins are disrupted, as they are in EDS, the result is structural laxity and disorganized tissue architecture. These mechanisms help explain many of the findings we observed in our study (Malfait et al. 2017; Smith et al. 2001; Salles Rosa Neto et al. 2024).

The most prominent pathological finding in our nvEDS cohort was a significantly higher prevalence of TMDs, affecting 64.3% of patients. This was primarily characterized by myofascial pain (57.14%) and joint hypermobility (21.4%). This finding is consistent with the known pathophysiology of EDS, where abnormalities in collagen‐V and tenascin‐X reduce the stiffness of joint capsules and ligaments, leading to TMJ instability (Malfait et al. 2017; Beighton et al. 1998). This instability increases the mechanical strain experienced by the jaw during daily movements, leading to muscle overuse and discomfort—patterns that fit well with the pain‐adaptation model of TMD (Malfait et al. 2017; Lepperdinger et al. 2021; Schiffman et al. 2014; Di Giacomo et al. 2018). Habits such as clenching or tongue pressing are likely to add additional strain (Schiffman et al. 2014). Although panoramic radiographs did not show increased TMJ remodeling, this acquisition protocol is limited for detecting early soft‐tissue changes. Future studies employing MRI or ultrasound would be better suited to assess subtler degenerative features (De Coster et al. 2005; Royer and Han 2022).

While periodontitis rates were similar to controls, gingivitis was more frequent in the nvEDS group than in the control group (85.71% vs. 56.67%). This may be related to reduced collagen stability and disorganized ECM from tenascin‐X deficiency, which could make gingival tissues more fragile and slower to heal (Mitakides and Tinkle 2017). Practical, everyday factors may also contribute; for example, pain from TMD can limit brushing, certain medications can cause dry mouth, and fatigue can impact hygiene consistency. Future studies that combine clinical data with measures of mucosal resilience and salivary flow would help clarify these patterns (Malfait et al. 2017; Kapferer‐Seebacher et al. 2021; Mitakides and Tinkle 2017; Järveläinen et al. 2009; Trombelli et al. 2018; Caton et al. 2018). Beyond soft tissue integrity, the ECM also plays a central role in tooth morphogenesis and root formation.

As for hard tissues, nvEDS patients more often had short roots (57.1%) and pulpal retraction (61.5%). There was no statistically significant difference in the history of dentofacial orthopedic treatment. We considered that once a quarter of a patient's teeth had been affected (i.e., 7 teeth or more), a clinical sign began to have clinical relevance and was probably not just a simple anatomical variation. We carried out some additional analyses of hypoplasia and pulpal retraction. Focusing only on patients with dental hypoplasia on at least 7 teeth, we found a statistical difference between the two groups with an OR of 11.6 [1.16; 116.42] 95% CI. There was also a statistically significant difference between the two groups for patients with at least 7 teeth with pulpal retraction, with an OR of 24 [2.47; 233.06] 95% CI (Table 3). The prevalence of short roots and pulpal constriction in nvEDS indicates that collagen and ECM dysregulation may interfere with normal dentinogenesis (Chen et al. 2019; Linde and Goldberg 1993). During dentin formation, type I collagen provides the framework for mineralization, while type V collagen helps control fibril size and organization. Tenascin‐X also supports matrix assembly and elasticity (Malfait et al. 2017; Lepperdinger et al. 2021; Järveläinen et al. 2009; Kapferer‐Seebacher et al. 2020). All of these matrix molecules interact in a highly coordinated manner in the morphogenesis of oral and dental structures. The disruptions in these processes could explain the root shortening and pulpal constriction we observed. This idea is supported by genetic studies linking mutations in collagen and dentin‐related genes (*DSPP*, *COL1A1*, *COL1A2*) with structural dental abnormalities (Li and Zhang 2022) and align with reports suggesting root hypoplasia may be a feature of cEDS (Kapferer‐Seebacher et al. 2020). It should be noted that, while shorter roots are often observed in nvEDS, vEDS has been linked with elongated root forms, suggesting subtype‐specific effects on odontogenesis. Animal models would be valuable for better understanding the molecular mechanisms underlying these oral manifestations. For example, type III collagen‐mutant mice display impaired type I collagen fibrillogenesis (Liu et al. 1997).

No significant differences were seen in pulp stones, dental agenesis, or DMFT scores. Due to the variability of these features within the general population and the small sample size, we cannot assume a true absence of effect. Similarly, the lack of difference in high‐arched palate may reflect natural variation or differences in sample composition across studies (Lepperdinger et al. 2021; Jannati et al. 2019; Jones 1984; Stappert et al. 2020). Larger cohorts are needed to explore these features with more confidence.

The most striking anatomical variation observed was the atrophy or absence of the lingual and inferior labial frenula observed in over 78% of nvEDS participants. Literature on this topic remains sparse and heterogeneous, largely because of differences in the composition of the studied populations and the evaluation methods employed (De Felice et al. 2001; Böhm et al. 2001). Studies based on clinical examinations tend to report higher prevalence than those based on questionnaires (Shankar et al. 2006; Savasta et al. 2021). Given the collagen‐I/III‐rich composition and stabilizing role of collagen‐V and tenascin‐X, such atrophy is biologically credible (Chen et al. 2019; Mills et al. 2020; De Castro Martinelli et al. 2014). Developing and validating a simple grading system for frenular atrophy would improve reproducibility.

A key strength of this study is that all nvEDS cases were molecularly confirmed and assessed using a standardized protocol, providing a reliable foundation for understanding subtype‐specific features. However, several limitations must be acknowledged. The single‐centre design and small sample size (14 nvEDS patients) limit statistical power and the generalizability of our findings. The use of panoramic imaging, while practical, also restricts the level of anatomical detail, particularly for the TMJ.

Most of the published literature on oral manifestations in EDS consists of small case series, often mixing subtypes without genetic confirmation, resulting in a fragmented and sometimes contradictory body of evidence (Kapferer‐Seebacher et al. 2021; Di Giacomo et al. 2018; Ferré et al. 2012; Savasta et al. 2021; Coster et al. 2005; Lepperdinger et al. 2022; Jensen and Storhaug 2011; Parrini et al. 2004). Our study contributes to a better understanding of the oral manifestations that may be linked to disease‐related tissue fragility. Stratifying oral findings by genetically confirmed EDS subtypes is crucial.

EDS encompasses 13 subtypes presenting distinct clinical patterns, and oral manifestations appear to mirror this diversity, highlighting how different molecular defects can shape distinct dental phenotypes. This diversity underscores the importance of analyzing subtypes separately rather than generalizing oral outcomes across the entire EDS spectrum (Ferré et al. 2012). To build a more robust evidence base, future research should prioritize multicenter collaborations to enable international data pooling, allowing for meaningful analysis of these rare and distinct subtypes.

Finally, our findings could support a biology‐informed, pragmatic approach to oral care in nvEDS. The high prevalence of TMDs calls for early screening and conservative management focused on controlling parafunctions and reducing joint strain. The observed gingival fragility suggests that short, frequent, and gentle hygiene sessions are preferable. Finally, dental anomalies like short roots necessitate adaptations in orthodontic or restorative treatments to minimize risks. Most importantly, oral findings such as frenular atrophy could serve as accessible clinical indicators, aiding in the early recognition of nvEDS and emphasizing the need for close collaboration between dental practitioners and medical specialists.

## 5 Conclusion

Our findings suggest that molecularly confirmed nvEDS patients present a characteristic pattern of oral manifestations. These include a high prevalence of TMDs, marked atrophy of the lingual and inferior labial frenula, and dental anomalies such as short roots and pulpal retraction. These manifestations are likely clinical expressions of the systemic connective tissue fragility inherent to EDS, stemming from underlying collagen and extracellular matrix abnormalities. These oral signs could serve as accessible indicators to aid in diagnosis, highlighting the need for close collaboration between dentists and physicians for comprehensive care. Given the limitations imposed by our small sample size, larger, multicenter studies are essential to validate these observations and determine their specificity across different EDS subtypes.

## Author Contributions

**Aude Grand:** writing – original draft, investigation, data curation. **Benjamin P. J. Fournier:** software, formal analysis. **Grégoire Chevalier:** investigation. **Karelle Benistan:** writing – review and editing, methodology, supervision. **François Côme Ferré:** conceptualization, writing – review and editing, supervision.

## Conflicts of Interest

The authors declare no conflicts of interest.

## Data Availability

The data that support the findings of this study are available on request from the corresponding author. The data are not publicly available due to privacy or ethical restrictions.
